# Comparative experience shapes sucrose preference through memory in *Drosophila*

**DOI:** 10.1186/s13041-025-01202-0

**Published:** 2025-04-10

**Authors:** Maximiliano Martinez-Cordera, Takaomi Sakai, Minoru Saitoe, Kohei Ueno

**Affiliations:** 1 Tokyo Metropolitan Institute of Medical Science, 2-1-6 Kamikitazawa, Setagaya-ku, Tokyo, 1568506 Japan; 2 Tokyo Metropolitan University, 1-1 Minami-Osawa, Hachioji-shi, Tokyo, 1920397 Japan

**Keywords:** Drosophila melanogaster, Feeding behavior, Sucrose preference, Mushroom body, Dopamine signaling

## Abstract

Selection of appropriate food is an ability that allows animals to make optimal foraging choices. However, the neural mechanisms that control this food selection remain unclear. The purpose of this study was to investigate the connection between memory and the feeding behavior of *Drosophila melanogaster* when two sucrose solutions with different concentrations are available. We placed flies into plates with 150 mM and 100 mM sucrose solutions and measured the preference for the 150 mM one. Flies preferred the 150 mM solution over the 100 mM when all 60 wells of the plate were filled with both solutions; this preference decreased when there were only 8 wells with food. Remarkably, prior exposure to a plate with all 60 wells filled with both solutions enhanced the preference for the 150 mM, even when there were only 8 wells with food. We found that the memory-related gene *rut* and the dopamine D1 receptor on the mushroom body were required to enhance the preference after the prior exposure. These findings show that memory acquired through experiencing both solutions is stored in the mushroom body optimizing the food selection process.

## Introduction

Adequate nutrition in a variety of environments is an important ability for animals. *Drosophila melanogaster* has developed as a valuable model organism for taste sensing and feeding behaviors [1, 2]. Previous studies have found that flies show acute sucrose discrimination, being able to distinguish between 10 mM and 10.25 mM sucrose solutions [3]. Interestingly, it has been suggested that this discrimination is achieved not only by promoting the intake of higher concentrations but also by inhibiting the ingestion of lower concentrations [3].

Furthermore, memory-related molecules such as *rut* or *dnc*, are necessary for this discriminative behavior [4]. Nevertheless, these molecules are also known to function in peripheral neurons other than the central brain [5, 6]. This raised uncertainty about whether flies rely primarily on memory to distinguish between sucrose concentrations or if sensory adaptation is a more significant factor.

Here, we aimed to address this gap by investigating the role of memory in feeding behavior when flies are presented with high or low sucrose solutions. We observed that when the feeding sites available are reduced, the discrimination of *Drosophila* for the high sucrose solution is also reduced. However, pre-exposure to both concentrations reinforced the discrimination even when the feeding site availability was subsequently reduced. Our results revealed that this selective feeding behavior relies on memory-related genes and dopamine signaling in the mushroom body, a key neural center for learning and memory in flies, suggesting that flies use memories of complex environmental information retained in the mushroom body and operated in a dopamine-dependent manner to make food choices, resembling associative memory processes.

## Materials and methods

### Fly strains and rearing

All experiments were performed on *Drosophila melanogaster* flies 2–5 days after eclosion. Using the Canton S strain as wild-type. As for memory deficient mutants, we use *rut*^*1*^ flies for *rutabaga* mutant [7], and *dumb*^*2*^ flies for dopamine D1 receptor mutant. OK107 was used for pan-mushroom body neurons GAL4 driver [8].

All fly stocks were raised on standard cornmeal medium at 25°C, in 60% relative humidity on a 12:12 h light/dark cycle.

### Two-choice test

The experimental methodology developed previous methods [9, 10]. Before the behavior test, flies were starved for 9 h in empty vials. Thirty to forty of those flies were introduced onto a 60-well micro-test plate (Nunc, Denmark) and allowed to feed. The wells in a micro-test plate were alternately empty or filled with sucrose solution, depending on the experiment. The higher-concentration sucrose solutions contained blue, and the lower-concentration solutions contained red food dye, and all solutions contained 1% agar. The blue dye was brilliant blue FCF (FUJIFILM Wako Pure Chemical Corporation, Japan) and the red dye was amaranth (SIGMA-Aldrich, USA), with final concentrations of 0.5 mg/ml and 0.25 mg/ml, respectively.

Naive experiments are measured by introducing the flies to the plate. In the experience procedure, the flies are placed on a plate with the short edge cut off (the cut-off side was covered with Parafilm), containing a solution without dye. After 3 min, the Parafilm was removed and connected to a plate with the same cut-off edges, and the flies were transferred to the plate, containing a solution with food dye, and allowed to feed for 12 min. All experiments were conducted under dark conditions limiting the flies’ visual information.

After feeding, flies were killed in a freezer and classified into four groups according to their abdominal color; blue (Nb), red (Nr), no staining (Nn), and Np represents the number of flies fed both blue and red solutions. The preference index for the higher-concentration sucrose was calculated as 100 x (Nb-Nr)/(Nb + Nr + Np). The percentage of Nn flies was smaller than 50% in all experiments. The percentage of Nn was about 20% or less in the naive experiments, but in the experienced procedure, the percentage was higher but never exceeded 50% because of the solution without dye in the first plate.

### Statistics

Normality tests were performed with the D’Agostino & Pearson test, the Anderson-Darling test or the Shapiro-Wilk test. In the case of the two-group test, an unpaired t-test was performed after confirming by the F-test that there was no significant difference in the variances. In the case of a three-group test, if both normality and variance tests were passed, a parametric one-way ANOVA and Dunnett or Tukey’s multiple comparisons test were performed (GraphPad Software, Inc., La Jolla, CA, USA). For all statistical analyses, significance levels were represented in figures using asterisks as follows: * *p* < 0.05, ** *p* < 0.01, *** *p* < 0.001, **** *p* < 0.0001, and ‘ns’ (not significant) for *p* > 0.05. Additionally, the exact *p*-values are provided below each significance marker.

## Results

### Reducing food location impairs selective feeding behavior

**Fig. 1:**
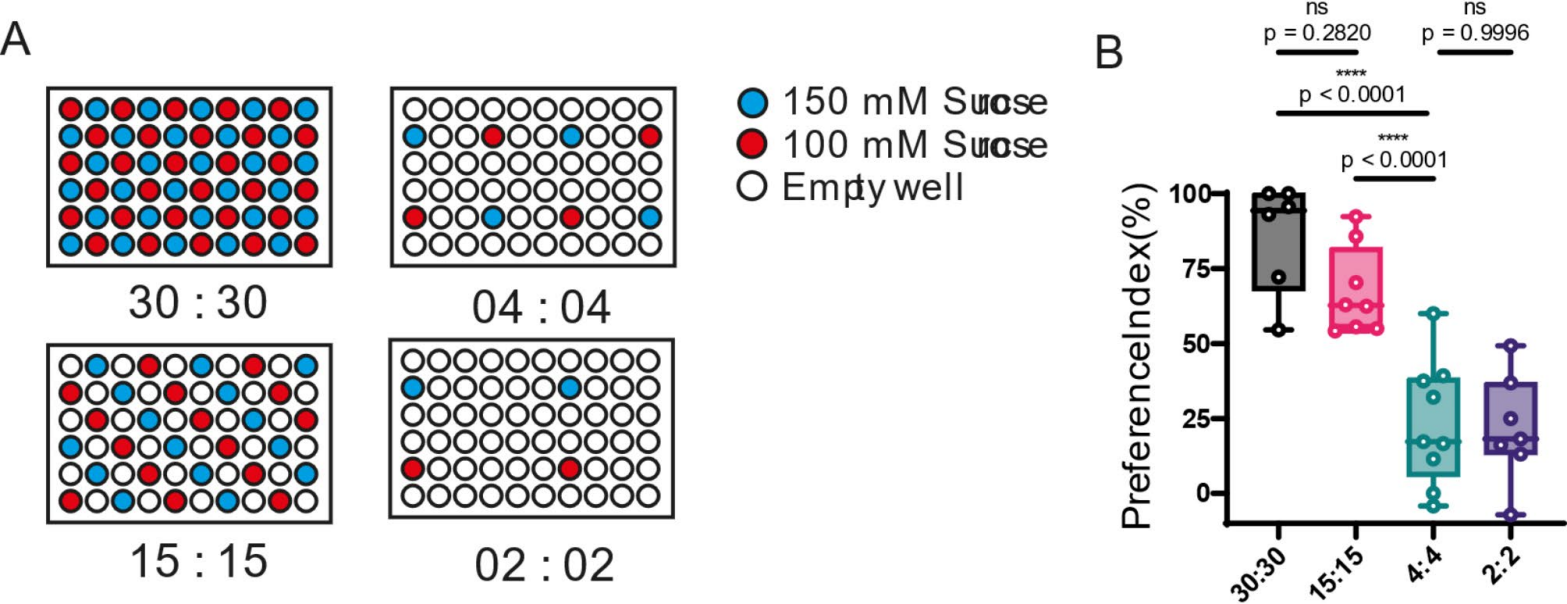
Effect of food availability on selective feeding in starved flies. (**A**) Experimental design showing 60-well plates with different configurations of 150 mM (blue) and 100 mM (red) sucrose solutions. (**B**) Preference index for 150 mM sucrose on each configuration. Median PI of 85.92% for 30:30, 67.32% for 15:15, 23.36% for 4:4, and 21.65% for 2:2 configurations. Each circle represents the preference index using about 40 flies. Box plots show median, 25th, and 75th percentiles and the whisker bars represent maximum and minimum values. Statistical significance was determined using a one-way ANOVA followed by a Dunnett post hoc testing

To investigate how flies selectively intake between two sucrose solutions, we chose 100- and 150-mM sucrose because previous electrophysiological studies demonstrated that these correspond to the midpoint of the dynamic range of gustatory receptor neuron responses in *Drosophila* [11, 12]. We placed flies in a setup with 60 well plates containing 150 mM and 100 mM sucrose solution, varying the number of wells containing each solution (Fig. 1A).The results demonstrated that when all 60 wells were filled with sucrose solutions (30:30), the preference index (PI) for 150 mM sucrose was 85.92%. However, reducing the number of wells to 15:15 significantly lowered the PI to 67.32%, and it further declined to 23.36% and 21.65% in the 4:4 and 2:2 configurations, respectively (Fig. 1B). These results resemble those observed in the 100 mM versus 10 mM setup, where the preference index declines as the well’s configuration changes from 30:30 to 4:4 [3]. Suggesting that the flies’ ability to exhibit selective feeding is impaired as the number of wells with solution decreases.

### Prior exposure to high-well availability increases PI

**Fig. 2:**
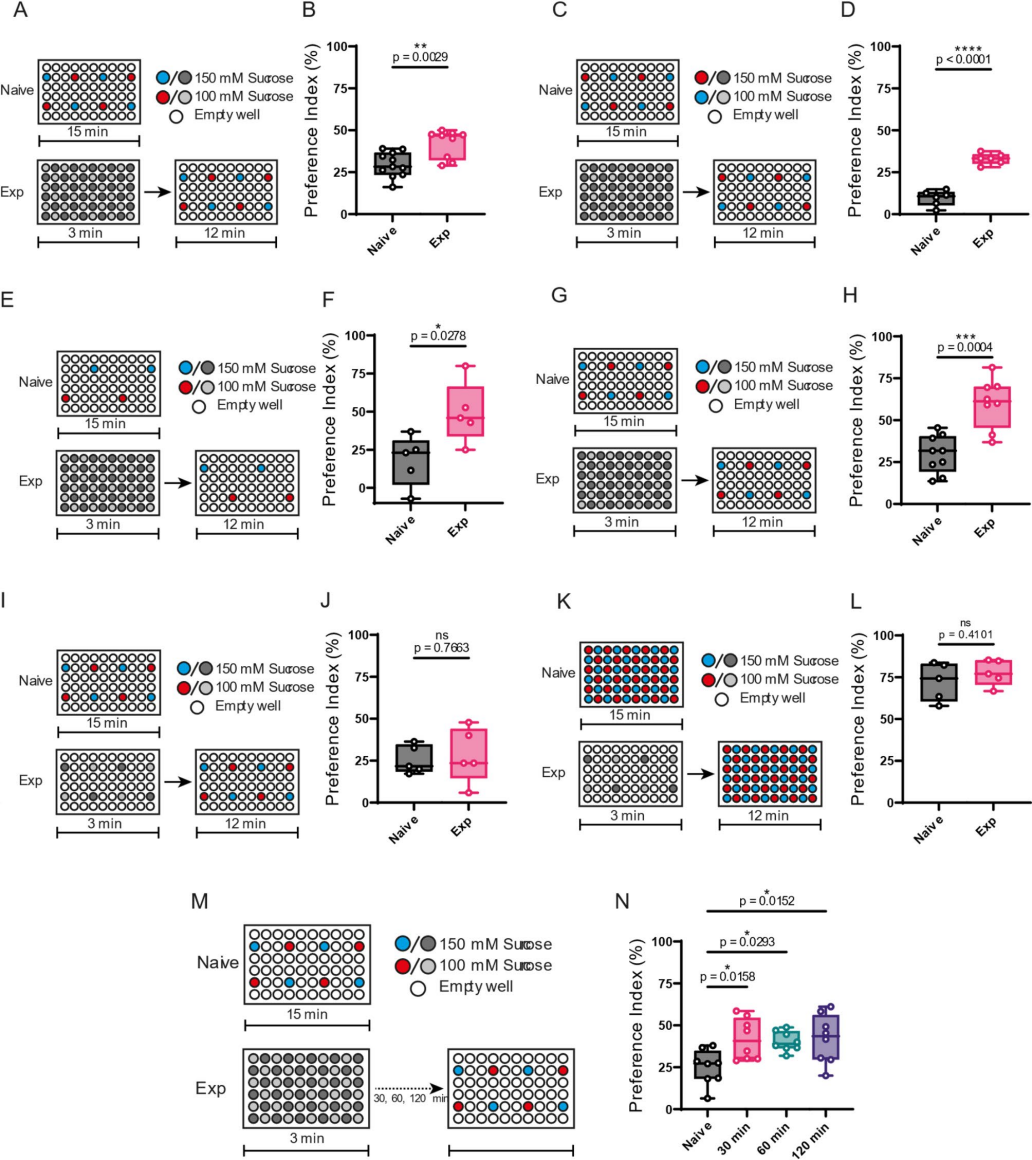
Previous experience enhances preference for 150 mM sucrose concentration on low-well configurations. (**A**, **C**, **E**, **G**, **I**, **K, M**) Schematic representations of the two-choice feeding assay for naive flies (top) and experienced flies (bottom). Naive flies were tested on a plate with a 4:4 configuration (test plate) for 15 min, while experienced flies underwent a pre-exposure period in a 30:30 configuration plate for 3 min followed by testing on a test plate unless otherwise stated. (**B**) PI for 150 mM solution on the test plate. Naive flies showed a median PI of 29.58% while experienced flies a 42.08%. (**C**) Representation of the feeding assay switching the red and blue dye. (**D**) Naive flies’ PI 9.59%, experienced flies’ PI 33.02%. (**E**) Representation of the feeding assay with a 2:2 configuration test plate. (**F**) Naive flies’ PI 17.86%, experienced flies’ PI 49.26% (**G**) Representation of the feeding assay with a 4:4 asymmetrical configuration test plate. (**H**) Naive flies’ PI 29.70%, experienced flies’ PI 60.08% (**I**) Representation of the feeding assay with a 4:4 configuration pre-exposure plate. (**J**) Naive flies’ PI 25.6%, experienced flies’ PI 25.15% (**K**) Representation of the feeding assay with a 4:4 configuration pre-exposure plate and a 30:30 configuration test plate. (**L**) Naive flies’ PI 72.31%, experienced flies’ PI 77.66% (**M**) Representation of the feeding assay with a 30-, 60-, and 120-minutes wait between the pre-exposure and the test. (**N**) Naive flies’ PI 25.14%, 30 min wait flies’ PI 41.88%, 60 min wait flies’ PI 40.41%, 120 min wait flies’ PI 41.97%. Each circle represents the preference index using about 40 flies. Box plots show median, 25th, and 75th percentiles and the whisker bars represent maximum and minimum values. Statistical significance was determined using a two-tailed Student’s T-test in B, D, F, H, J and L. Statistical significance in N was determined using a one-way ANOVA followed by a Dunnett post hoc testing

Previous studies showed that increasing the travel time between food encounters in sparse environments reduce foraging behavior [13]. We assume that the decline in the PI is due to the reduced encounter frequency result of the reduction of sucrose wells. To test this idea, we introduced flies into a 30:30 configuration plate for 3 min to ensure sufficient opportunities to encounter different sucrose wells and then transferred to a 4:4 configuration (Fig. 2A). The results showed that flies previously exposed to the 30:30 configuration exhibited a significantly higher PI of 41% in the subsequent 4:4 configuration, compared to the PI of 25% for flies without prior exposure (Fig. 2B). The effect of prior experience was observed even when the food dye assignments were reversed (Fig. 2C and D). A similar effect was observed in the 2:2 configuration, where prior exposure also increased PI (Fig. 2E and F).

To determine if the enhancement in the PI was due to the solution’s location, since the same wells contained similar solutions in both configurations. We exchanged the location of the solutions in the 30:30 plate (Fig. 2G). Notably, this increased PI was maintained even when the locations of the sucrose solutions in the 30:30 were exchanged (Fig. 2H), suggesting that the effect was not dependent on the specific spatial arrangement of the wells.

Nevertheless, pre-exposing flies to a 4:4 configuration instead of the 30:30 configuration (Fig. 2I and K), made no difference in the PI compared with flies without previous exposure when they were transferred to a test plate with either a in a 4:4 (Fig. 2J) or a 30:30 configuration (Fig. 2L). These results suggest that sufficient opportunities to encounter sucrose solutions are critical for the increment in PI and that flies may further retain this information to guide their selective feeding behavior.

### Memory retention supports long-term selective feeding

To confirm that flies use information about their surrounding feeding environment—potentially relying on some form of memory—to improve the selective feeding, we extended the time interval between their transfer from the 30:30 configuration to the 4:4 configuration (Fig. 2M). Remarkably, flies maintained a high PI immediately after transfer and 30, 60, and even 120 min later (Fig. 2N). These findings further support our hypothesis that memory plays a role in guiding selective feeding behavior. However, we could not test longer intervals, as prolonged starvation led to fly mortality.

### Selective behavior requires multiple sucrose concentrations

**Fig. 3:**
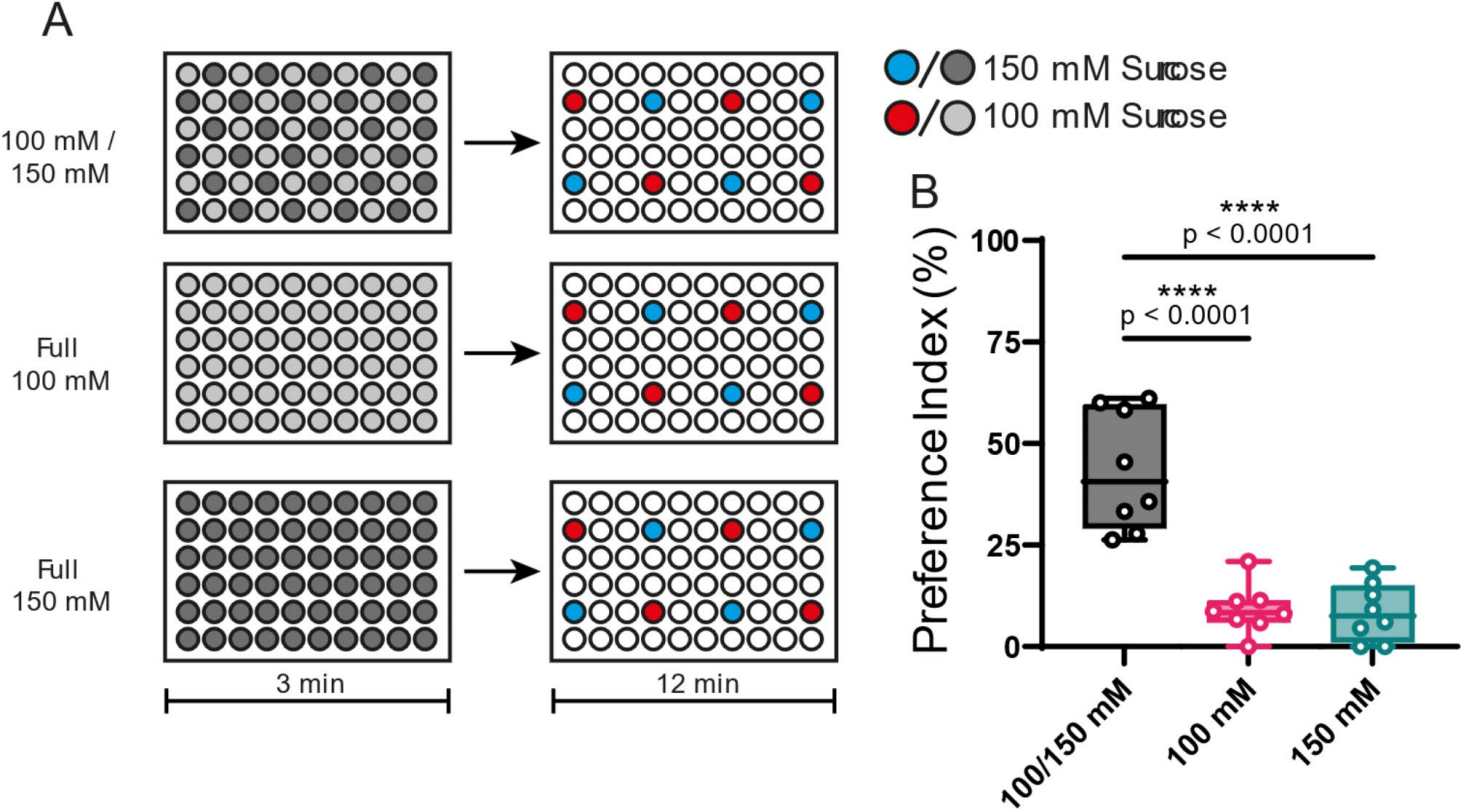
The enhancement selective feeding depends on training with multiple sucrose concentrations. (**A**) Schematic representation of training plates’ configuration. Flies were pre-exposed to plates with a 30:30 configuration or a 60:0 configuration with only one type of sucrose solution, either 100 mM (light gray) or 150 mM (dark gray) for 3 min. Testing was conducted on plates with a 4:4 configuration under identical conditions for all groups. (**B**) Flies which experienced both concentrations showed a PI of 43.71%, flies which experienced only one solution showed a PI of 9.08% and 8.43% respectively. Each circle represents the preference index using about 40 flies. Box plots show median, 25th, and 75th percentiles and the whisker bars represent maximum and minimum values. Statistical significance was determined using one-way ANOVA followed by Tukey post hoc testing

Next, to determine if this enhanced selectivity could be explained by the sensory processing of the previous experience either habituation to the higher sucrose concentration of sensitization to the lower concentration or by more specific aspects of their surrounding feeding environment, we prepared three types of 30:30 plates: one containing 150 mM and 100 mM sucrose solutions and two others containing either 150 mM or 100 mM sucrose exclusively (Fig. 3A). The results showed that flies exhibited a high PI only when presented with the plate containing both sucrose concentrations (Fig. 3B), suggesting that multiple concentration options are crucial for the observed enhancement in the selective feeding behavior.

### Memory mutants fail to exhibit selective feeding

**Fig. 4:**
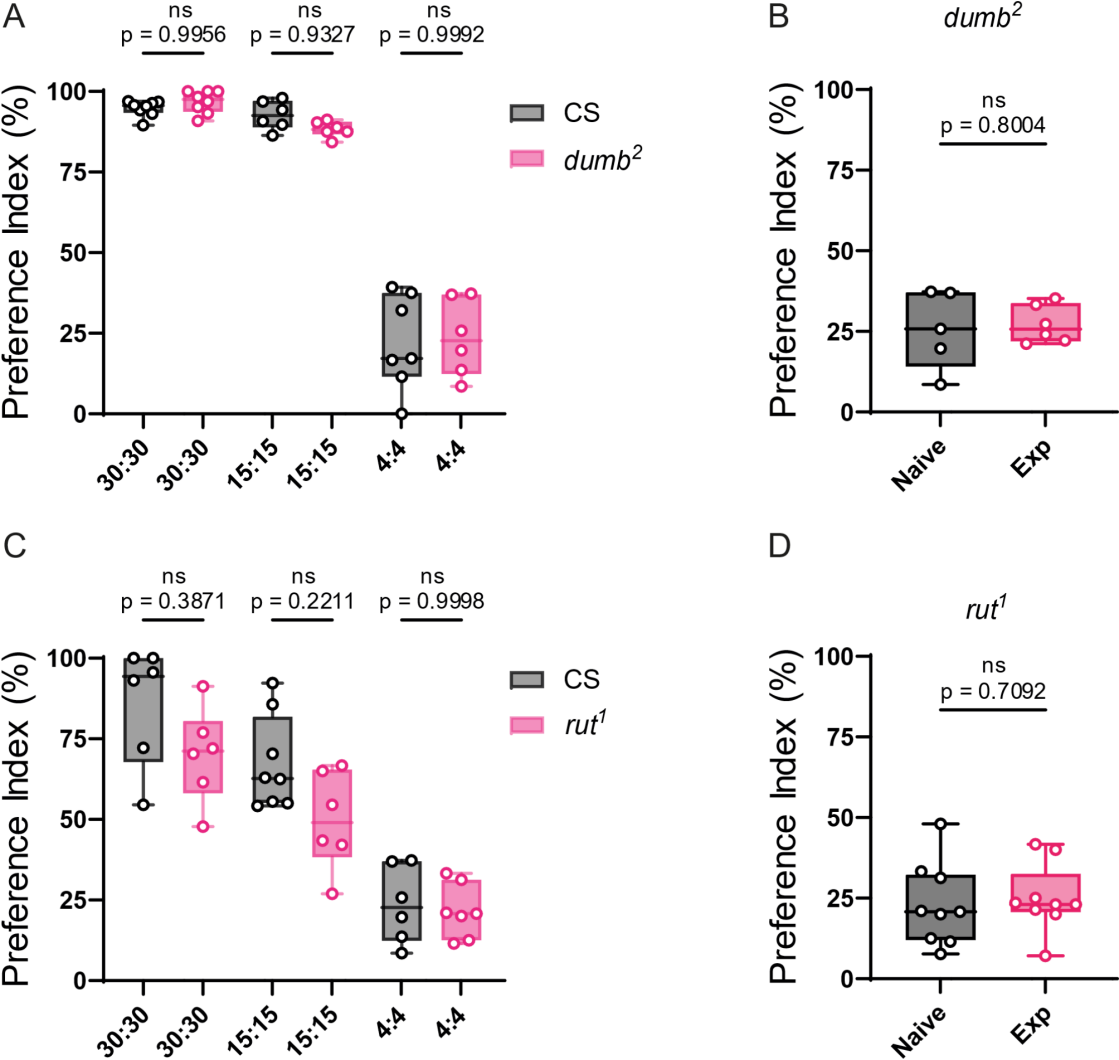
Short term memory mutants do not show enhancement of selective feeding after pre-exposure. (**A**) Comparison of PI for 150 mM solution on 30:30, 15:15, and 4:4 configurations between CS flies and *dumb*^*2*^ flies. CS flies showed a median PI of 94.78%, 92.66%, and 22.05% respectively while *dumb*^*2*^ flies’ PI were 96.81%, 88.3%, and 23.64% respectively. (**B**) Preference index of *dumb*^*2*^ mutant flies, naive and with pre-exposure (25.64% and 27.22% respectively). (**C**) Comparison of PI for 150 mM solution on 30:30, 15:15, and 4:4 configurations between CS flies and *rut*^*1*^ flies. CS flies showed a median PI of 85.92%, 67.32%, and 22.95% respectively while *rut*^*1*^ flies’ PI were 85.92%, 49.79%, and 21.5% respectively (**D**) Preference index of *rut*^1^, naive and with pre-exposure (22.91% and 24.99% respectively). Each circle represents the preference index using about 40 flies. Box plots show median, 25th, and 75th percentiles and the whisker bars represent maximum and minimum values. Statistical significance was determined using a two-tailed Student’s t-test

**Fig. 5:**
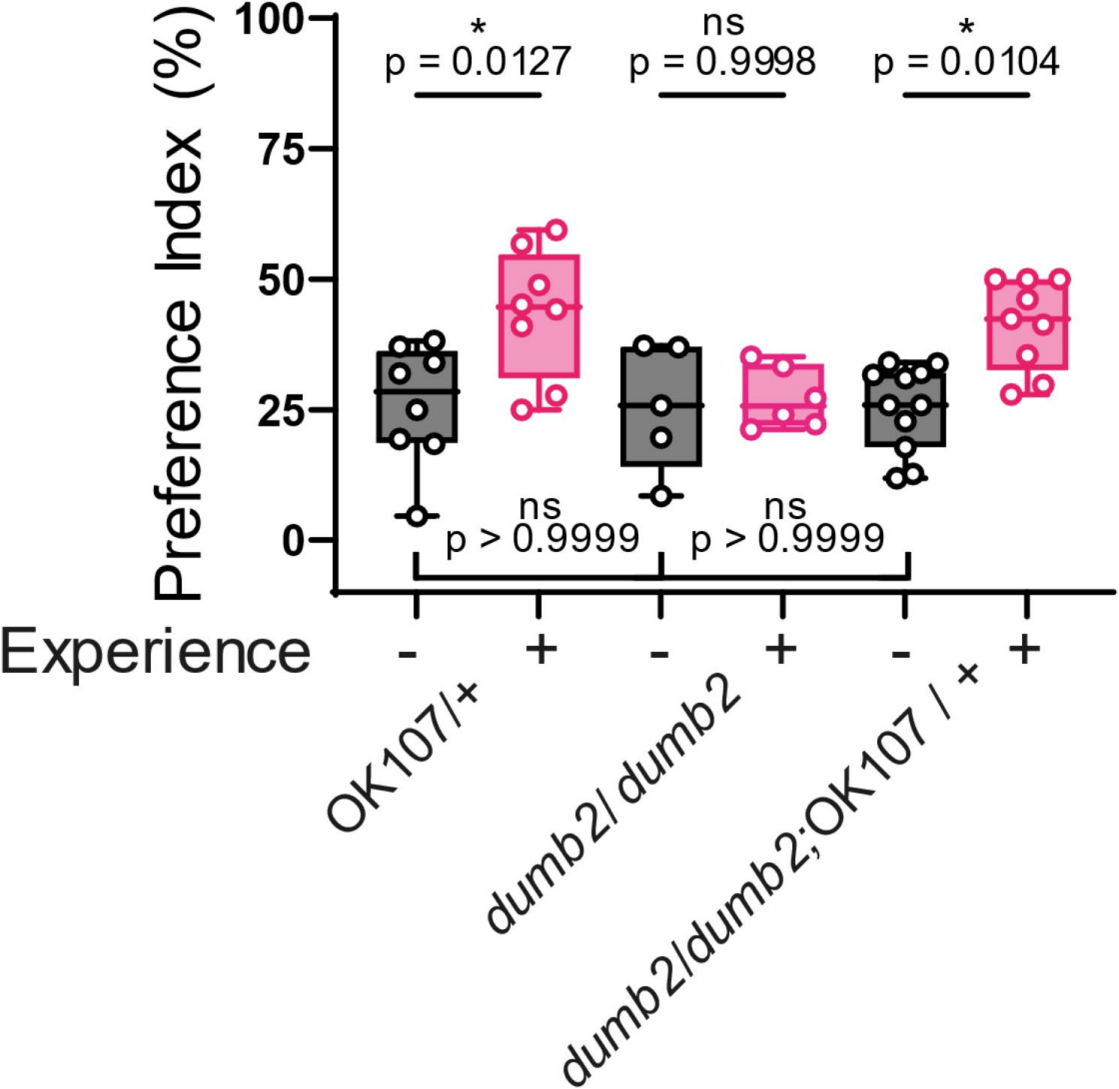
Rescue of *dumb*^*2*^ expression in the mushroom body restores experience-induced enhancement of feeding selectivity. PI for the 150 mM sucrose solution, black boxes represent naive flies, and pink boxes represent experienced flies. The first two boxes represent OK107 flies as genetic control showing a PI of 26.07% and 43.53%. The second two boxes represent *dumb*^*2*^ mutants, which show a PI of 25.64% and 27.22% respectively. The final two boxes represent the rescued genotype (*dumb*^*2*^*/dumb*^*2*^; OK107), which shows PIs of 25.41% and 41.44%. Each circle represents the preference index using about 40 flies. Box plots show median, 25th, and 75th percentiles and the whisker bars represent maximum and minimum values. Statistical significance was determined using one-way ANOVA followed by Tukey post hoc testing

To further investigate the role of memory in selective feeding, we conducted experiments using the memory mutants *rut*^*1*^ and *dumb*^*2*^ [7, 14, 15] which present similar PI scores when presented to the 30:30, 15:15, and 4:4 configuration to the PI presented by wild-type flies (Fig. 4A and C).　Flies of these mutants were exposed to the 30:30 configuration and then transferred to the 4:4 configuration. Unlike wild-type flies, *rut*^*1*^ and *dumb*^*2*^ mutants failed to increase the PI in the 4:4 setup after prior exposure to the 30:30 plate (Fig. 4B and D). To confirm that these memory-related molecules indeed function in the central brain, we performed rescue experiments using the *dumb*^*2*^ mutant. In the *dumb*^*2*^ mutant, a piggyBac containing a UAS sequence activated by GAL4 is inserted into the first intron, which suppresses the endogenous expression of dopamine D1 receptor. Thus, crossing this mutant with a GAL4 driver can restore dopamine D1 receptor expression [15]. For this rescue experiment, we crossed the *dumb*^*2*^ mutant with the pan-mushroom body GAL4 driver OK107 to generate *dumb*^*2*^/*dumb*^*2*^; OK107/+ flies. These flies exhibited a significant higher PI in the 4:4 configuration after previous exposure to the 30:30 configuration without any effect in the control experiments (Fig. 5), suggesting that selective feeding behavior requires dopamine signaling in the mushroom body neurons.

## Discussion

*Drosophila melanogaster* exhibits acute selectivity for different sucrose concentrations, but this selectivity diminishes as the number of available feeding sites decreases [3]. In this study, we demonstrate that *Drosophila*’s feeding selectivity is enhanced when the flies are first presented with a high-opportunity feeding environment before the options are reduced. This increase in selective feeding persists for over two hours after the previous experience. Moreover, this enhancement requires the flies to experience both sucrose concentrations, as prior exposure to only one concentration failed to increase preference in the 4:4 configuration. These findings indicate that sugar selectivity in flies is not due to simple habituation or sensitization at the level of taste receptor cells but is a sophisticated brain function performed by memory formation and recall in the central brain.

We found that this experience-dependent selectivity is impaired in the dopamine D1 receptor mutant and can be restored by expressing this receptor gene in the mushroom body. Previous findings indicate that a subset of dopaminergic neurons innervating mushroom body respond to sugar stimulation [16, 17]. This suggests that these neurons play a critical role in processing feeding-related experiences and guiding future food choices based on memory. The mushroom body is a well-established center for associative memory in *Drosophila* and sugar information is also represented in the mushroom body neurons [18], and our results further support its involvement in feeding behavior optimization through memory-dependent mechanisms.

Interestingly, we found that flies can retain information about prior sucrose exposure for more than 120 min. This retention period aligns with medium-term memory in olfactory learning paradigms [19] and previous study demonstrates that mutant of medium-term memory also impairs sugar selectivity [4], suggesting that similar neural mechanisms may underlie both associative olfactory learning and food experience mechanism.

Our findings also highlight the importance of environmental complexity in shaping feeding decisions. Flies pre-exposed to a 30:30 sucrose configuration exhibited an enhanced preference even when subsequently tested in a 4:4 or 2:2 configuration. This suggests that *Drosophila* integrate previous sugar experiences into their decision-making process, enabling them to optimize their feeding choices under changing conditions. Moreover, this enhancement was not dependent on the spatial location of sucrose wells, indicating that the flies retained information about the sucrose concentrations rather than the physical arrangement of the food sources.

In contrast, our results indicate that the preference observed in high-opportunity environments (30:30 and 15:15) is primarily driven by sensory processing rather than memory, since both *rut*^*1*^ and *dumb*^*2*^ mutants exhibited preference indices comparable to wild-type flies without prior exposure. Interestingly, previous studies demonstrated that memory mutants including *rut*^*1*^ show significantly reduced sucrose preference even in a 30:30 condition when the sucrose concentrations were 30mM and 40mM [4]. This discrepancy may indicate that the relative contribution of memory and sensory processing depends on the magnitude of the sucrose concentration difference. When the contrast is small (e.g., 30 mM vs. 40 mM), memory may influence the sucrose preference. However, when the contrast is increase (100 mM vs. 150 mM), real-time gustatory processing may be sufficient to drive food selection.

Overall, this study reveals that memory acquisition and recall significantly contribute to feeding discrimination in *Drosophila*. The ability to store and retrieve information about prior sucrose exposure allows flies to optimize food selection in dynamic environments. This underscores the adaptive value of memory in shaping foraging strategies and highlights potential parallels between feeding-related memory processes in *Drosophila* and other animals. Further investigations into the underlying neural circuits will provide deeper insights into the relationship between experience, memory, and decision-making in feeding behavior.

## Data Availability

No datasets were generated or analysed during the current study.

## References

[1] Scott K. Gustatory processing in drosophila melanogaster. Annu Rev Entomol. 2018;63:15–30. 29324046 10.1146/annurev-ento-020117-043331

[2] Miroschnikow A, Schlegel P, Pankratz MJ. Making feeding decisions in the drosophila nervous system. Curr Biol. 2020;30(14):R831–40. 32693083 10.1016/j.cub.2020.06.036

[3] Shimada I, Nakao M, Kawazoe Y. Acute differential sensitivity and role of the central nervous system in the feeding behavior of drosophila melanogaster. Chem Senses. 1987;12(3):481–90.

[4] Motosaka K, Koganezawa M, Narikawa S, Furuyama A, Shinozaki K, Isono K, Shimada I. Cyclic AMP-dependent memory mutants are defective in the food choice behavior of drosophila. J Comp Physiol Neuroethol Sens Neural Behav Physiol. 2007;193(2):279–83. 10.1007/s00359-006-0200-z 17180701

[5] Engel JE, Wu CF. Neurogenetic approaches to habituation and dishabituation in drosophila. Neurobiol Learn Mem. 2009;92(2):166–75. 18765288 10.1016/j.nlm.2008.08.003 PMC2730516

[6] Kuromi H, Kidokoro Y. Tetanic stimulation recruits vesicles from reserve pool via a cAMP-mediated process in drosophila synapses. Neuron. 2000;27(1):133–43. 10939337 10.1016/s0896-6273(00)00015-5

[7] Feany MB. Rescue of the learning defect in Dunce, a drosophila learning mutant, by an allele of Rutabaga, a second learning mutant. Proc Natl Acad Sci U S A. 1990;87(7):2795–9. 2157213 10.1073/pnas.87.7.2795 PMC53777

[8] Aso Y, Grubel K, Busch S, Friedrich AB, Siwanowicz I, Tanimoto H. The mushroom body of adult drosophila characterized by GAL4 drivers. J Neurogenet. 2009;23(1–2):156–72. 19140035 10.1080/01677060802471718

[9] Ueno K, Kohatsu S, Clay C, Forte M, Isono K, Kidokoro Y. Gsalpha is involved in sugar perception in drosophila melanogaster. J Neurosci. 2006;26(23):6143–52. 16763022 10.1523/JNEUROSCI.0857-06.2006 PMC6675175

[10] Tanimura T, Isono K, Takamura T, Shimada I. Genetic dimorphism in the taste sensitivity to Trehalose InDrosophila melanogaster. J Comp Physiol? A. 1982;147(4):433–7.

[11] Hiroi M, Meunier N, Marion-Poll F, Tanimura T. Two antagonistic gustatory receptor neurons responding to sweet-salty and bitter taste in drosophila. J Neurobiol. 2004;61(3):333–42. 15389687 10.1002/neu.20063

[12] Ueno K, Kidokoro Y. Adenylyl cyclase encoded by AC78C participates in sugar perception in drosophila melanogaster. Eur J Neurosci. 2008;28(10):1956–66. 19046378 10.1111/j.1460-9568.2008.06507.x

[13] Koganezawa M, Hara H, Hayakawa Y, Shimada I. Memory effects on scale-free dynamics in foraging drosophila. J Theor Biol. 2009;260(3):353–8. 19559713 10.1016/j.jtbi.2009.06.018

[14] Livingstone MS, Sziber PP, Quinn WG. Loss of calcium/calmodulin responsiveness in adenylate cyclase of Rutabaga, a drosophila learning mutant. Cell. 1984;37(1):205–15. 6327051 10.1016/0092-8674(84)90316-7

[15] Kim YC, Lee HG, Han KA. D1 dopamine receptor dDA1 is required in the mushroom body neurons for aversive and appetitive learning in drosophila. J Neurosci. 2007;27(29):7640–7. 17634358 10.1523/JNEUROSCI.1167-07.2007 PMC6672866

[16] Liu C, Placais PY, Yamagata N, Pfeiffer BD, Aso Y, Friedrich AB, Siwanowicz I, Rubin GM, Preat T, Tanimoto H. A subset of dopamine neurons signals reward for odour memory in drosophila. Nature. 2012;488(7412):512–6. 22810589 10.1038/nature11304

[17] Huetteroth W, Perisse E, Lin S, Klappenbach M, Burke C, Waddell S. Sweet taste and nutrient value subdivide rewarding dopaminergic neurons in drosophila. Curr Biol. 2015;25(6):751–8. 25728694 10.1016/j.cub.2015.01.036 PMC4372253

[18] Kirkhart C, Scott K. Gustatory learning and processing in the drosophila mushroom bodies. J Neurosci. 2015;35(15):5950–8. 25878268 10.1523/JNEUROSCI.3930-14.2015 PMC4397596

[19] Margulies C, Tully T, Dubnau J. Deconstructing memory in drosophila. Curr Biol. 2005;15(17):R700–713. 16139203 10.1016/j.cub.2005.08.024 PMC3044934

